# Localized intramural reentry confined within the ventricular septum in lamin cardiomyopathy

**DOI:** 10.1016/j.hrcr.2022.09.010

**Published:** 2022-09-22

**Authors:** Jake Martinez, Rong Bai, Marwan Bahu, Michael F. Morris, J. Peter Weiss, Roderick Tung

**Affiliations:** The University of Arizona College of Medicine-Phoenix, Banner University Medical Center, Phoenix, Arizona

**Keywords:** Ventricular tachycardia, Interventricular septum, Reentry, Septal perforator vein, Lamin cardiomyopathy, Midmyocardial, Intramural

**Key Teaching Points**

• *LMNA* mutations are characterized by septal and basal anterior wall substrate, which can be detected by microcatheters placed within the distal tributaries of the coronary venous system.
• Intramural recordings enhance the ability to characterize both the arrhythmogenic substrate and ventricular tachycardia circuit in cases where diastolic components are absent from surface recordings.
• Localized reentry can occur completely confined inside in the septum.

## Introduction

High-resolution electroanatomic mapping systems have greatly improved the ability to characterize the size and dimensions of the reentrant circuit responsible for human ventricular tachycardia (VT). The minimal dimension of critical isthmus regions may be less than 1 cm in more than 25% of circuits mapped.^1^ Despite advanced, detailed simultaneous epicardial and endocardial mapping, detection of intramural circuit components remains challenging. Epicardial mapping through coronary venous branches has gained popularity owing to refinement of mapping catheters and novel use of transcoronary venous ethanol. The septal venous perforators (SPV) that originate from the anterior interventricular vein (AIV) provides access into the basal superior intraseptal region of the left ventricle and may inform the most optimal vantage point for ablation.^2^ Anteroseptal substrates are among the most challenging in patients with dilated cardiomyopathies who present for VT ablation and the mechanisms are incompletely elucidated. Here, we report a case of a localized VT circuit within a midmyocardial septal substrate that was completely characterized by activation and entrainment mapping from 1 recording bipole pair within an SPV in a patient with lamin cardiomyopathy.

## Case report

**Figure 1:**
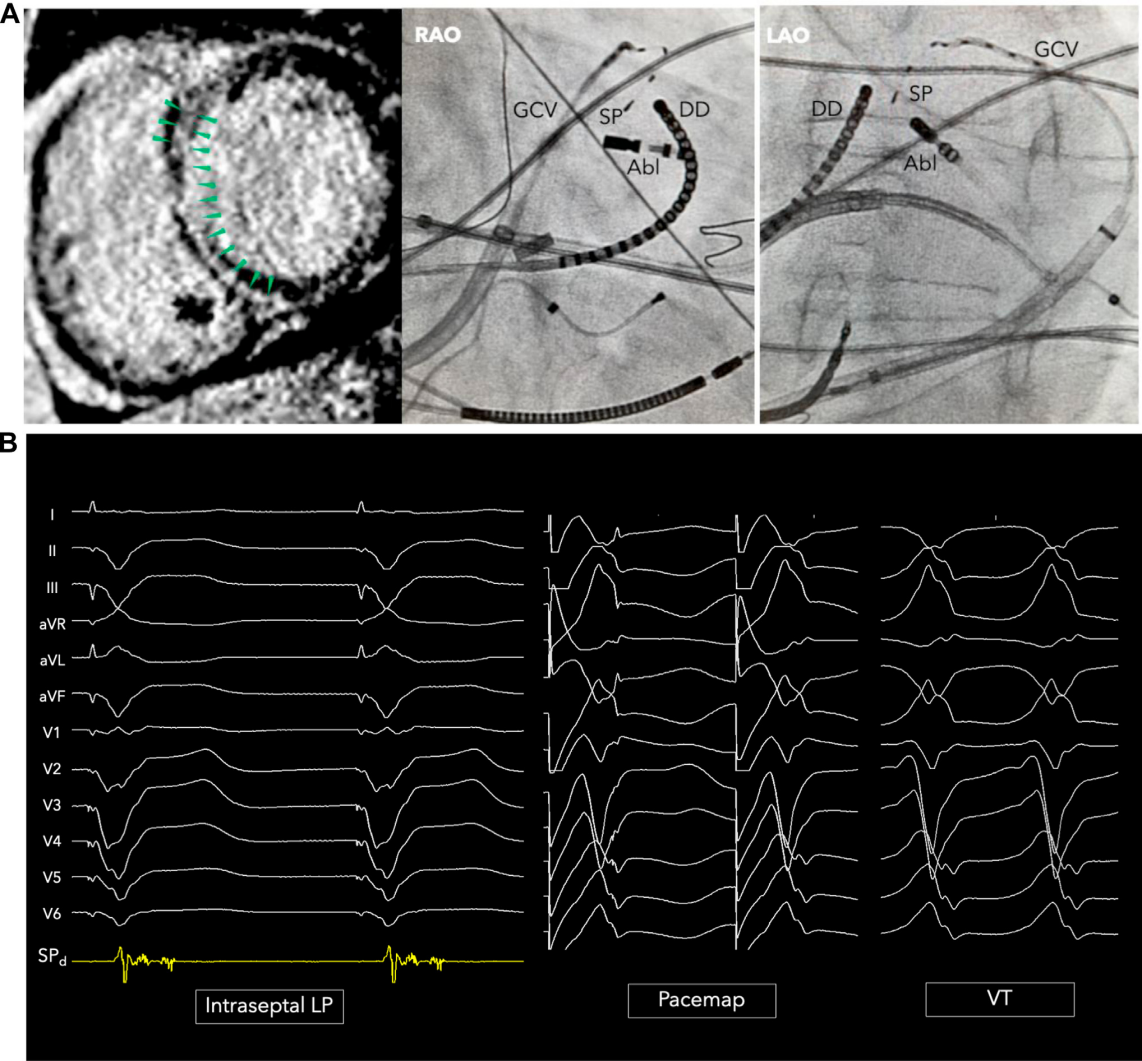
**A:** Midmyocardial septal scar and mapping catheters positioned to map ventricular tachycardia (VT) activation. A duodecapolar catheter (DD) was positioned along the right ventricular septum and ablation catheter (Abl) on the left ventricular septum via transseptal approach. A 2F mapping catheter was positioned with the 2 most distal electrodes within the septal perforating vein (SPV). **B:** An intraseptal fractionated late potential is recorded from within the SPV, which yields a near-perfect pace map match for the clinical induced VT with stimulus-to-QRS latency. GCV = great cardiac vein; LAO = left anterior oblique; SP = septal perforator vein; RAO = right anterior oblique.

A 73-year-old man with history of dilated cardiomyopathy and left ventricular (LV) ejection fraction of 39%, complete heart block status post permanent pacemaker placement 15 years ago with upgrade to biventricular implantable cardioverter-defibrillator 2 years ago, presented with recurrent episodes of VT and appropriate implantable cardioverter-defibrillator therapies despite antiarrhythmic drug therapy with sotalol. Cardiac magnetic resonance imaging showed midmyocardial delayed enhancement with full-length midmyocardial scar from the basal anteroseptum to inferoseptum (Figure 1A). He underwent initial ablation where septal VTs were mapped and targeted from the right ventricle. Owing to increasing VT burden with appropriate device therapies, including multiple shocks, he was referred for repeat ablation.

In anticipation of mapping a septal VT, a linear multielectrode catheter (Livewire; Abbott, Abbott Park, IL) was placed in the septal right ventricular outflow tract (RVOT) and an ablation catheter (FlexAbility; Abbott) was positioned on the LV basal anterior septum via transseptal approach. A diagnostic 2F microcatheter (EPstar, 1.5 mm distal electrode, 1.3 mm proximal electrodes, 5-5-5 mm spacing; Baylis Medical, Montreal, QC, Canada) was placed into the distal coronary sinus to record within the AIV and SPV (Figure 1A). During sinus rhythm, the Baylis catheter placed in the AIV/SPV recorded a low-voltage fractionated and split late potential with discrete isoelectric segment. Pace mapping from the AIV and SPV showed a near-perfect QRS match with stimulus-to-QRS latency for the clinical VT (Figure 1B).

**Figure 2:**
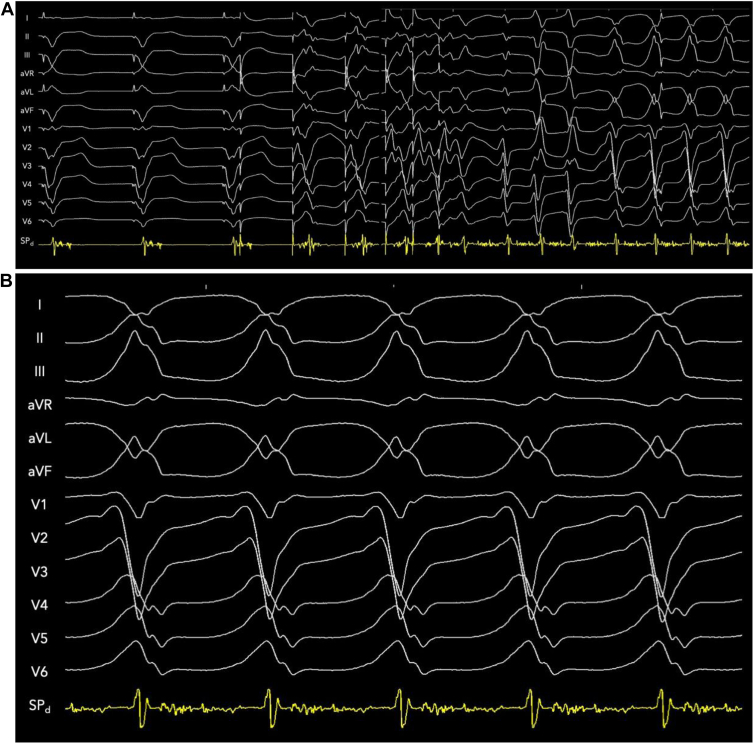
**A:** Continuous strip from sinus rhythm with induction of ventricular tachycardia (VT) with triple extrastimuli (500-300-260-250 ms) to demonstrate constant gain and minimal baseline noise. Five beats of the drive cycle have been removed. The intraseptal late potential (SPd) recorded during pacing rhythm become pandiastolic during VT. **B:** Twelve-lead electrocardiogram of VT (tachycardia cycle length 360 ms) with septal perforating vein recording spanning entirety of diastole at 150 mm/s.

**Figure 3:**
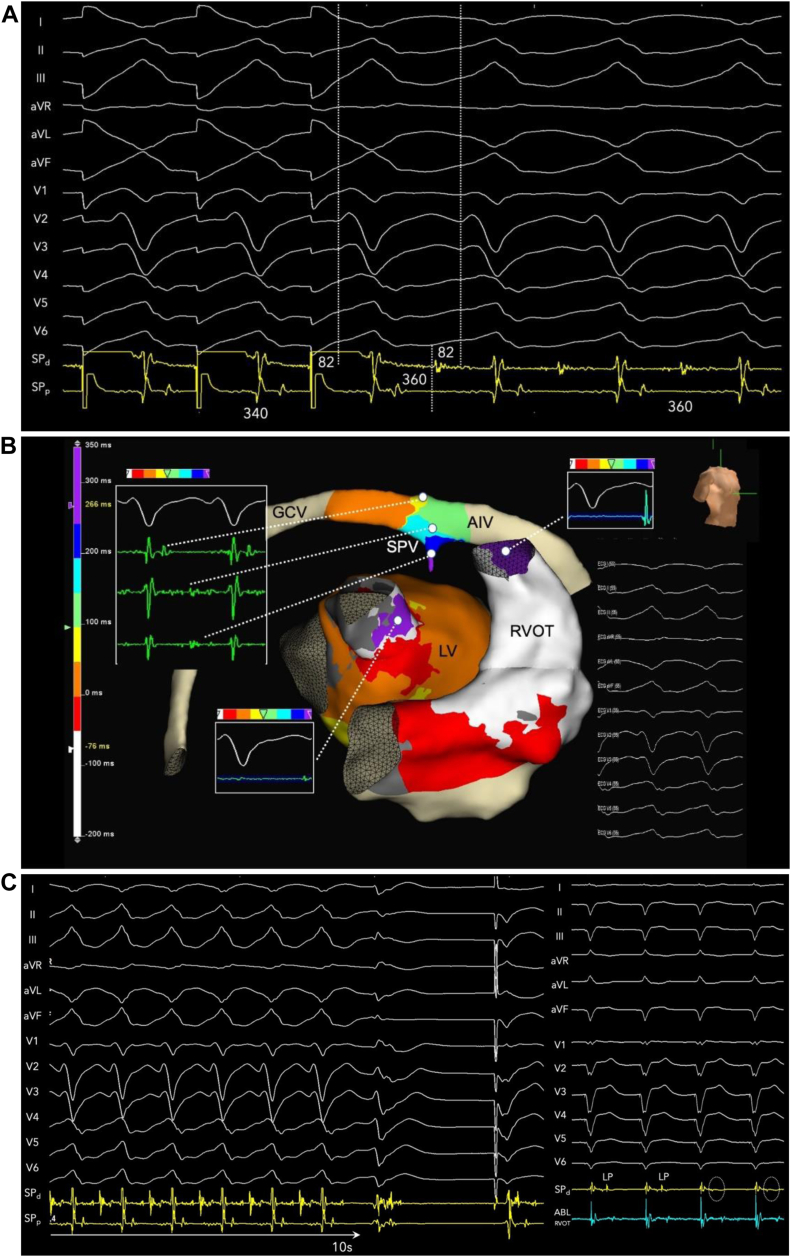
Activation mapping (**A**) and concealed entrainment (**B**) of ventricular tachycardia with termination during radiofrequency delivery (**C**). The intraseptal late potential is finally eliminated and dissociated after ablation from the right ventricular side of the septum. AIV = anterior interventricular vein; GCV = great cardiac vein; LV = left ventricle; RVOT = right ventricular outflow tract; SPV = septal venous perforators.

VT was induced with programmed triple extrastimuli from the RVOT. The local intraseptal fractionated and late electrogram exhibited pandiastolic activation spanning the entirety of diastole during VT. Figure 2A shows the continuous tracing from paced rhythm during sinus into VT from triple extrastimuli (last 5 beats of drive removed) at a constant gain to illustrate that diastolic activation during VT is not noise, given the clean baseline. Figure 2B shows VT with instraseptal recording at 150 mm/s. Activation mapping was performed from the right ventricular (RV) endocardium, LV endocardium, epicardially via great cardiac vein / AIV, and intramurally via SPV. Focal exit activation was observed on both LV (-45 ms pre-QRS) and RV endocardium (at QRS onset) (Figure 3A). Entrainment from a slightly more proximal position within the SPV showed concealed fusion with postpacing interval exactly matching VT cycle length and the S-V interval equal to the electrogram-QRS interval measured from SP_d_, proving critical circuitry confined within the interventricular septum (Figure 3B).

Ablation was performed from the corresponding LV endocardial surface and terminated VT within 10 seconds (Figure 3C, left). Additional ablations were performed but failed to alter the split and late potentials. Therefore, ablation was then performed from the septal RVOT with eventual elimination of late potentials and dissociated activity (Figure 3C, right). The clinical VT was rendered noninducible.

A second VT with left bundle branch block morphology with V_4_ transition and indeterminate axis with lead 2 more positive than lead 3, consistent with a midseptal right-sided breakthrough, was then identified, with activation mapping confirming the exit site inferior to the prior lesions. The periaortic region was extensively targeted without termination of VT and 2 sequential injections of 4 mL of 100% ethanol were infused into the septal perforator vein with termination of targeted VT 61 seconds after ethanol injection. Owing to the small size of the perforator vein with occlusion upon cannulation using coronary guide catheter (no contrast reflux), a balloon technique was not implemented. However, VT remained reinducible at a faster rate and therefore additional ablation were performed at 50 W from the LV and RV septum. VT has not recurred for 9 months at the time of this report.

## Discussion

Catheter ablation for drug-refractory VT in patients with dilated cardiomyopathies, particularly in those with genetic mutations such as *LMNA* and *TTN*, are associated with VT recurrence rates that are among the highest in structural heart disease.^3^, ^4^, ^5^ The advent of improved genetic testing has led to improved identification of likely pathologic variants associated with dilated cardiomyopathy and VT, allowing for better understanding of the substrate and outcomes based on the specific etiology of dilated cardiomyopathy. Patients with pathologic genetic variants, when compared to those without pathologic variants, were shown to have significantly lower 2-year VT-free survival and increased cardiac and all-cause mortality.^5^ *LMNA* variants in particular have been shown to have high rates of arrhythmia recurrence, progression of cardiomyopathy to end-stage heart failure, and high mortality.^3^

Lamin cardiomyopathy classically involves the septum and periaortic region and seems to be predominantly related to reentry in and around regions of scar. Periaortic VTs have been increasingly described with regard to reentrant mechanism and circuit characteristics.^6^ Midmyocardial scar patterns are associated with adverse outcomes and may require adjunctive strategies such as bipolar ablation with half normal saline as irrigant. Although the method and yield of intramural mapping via coronary veins have been described,^2^^,^^7^^,^^8^ complete activation mapping incorporating midmyocardial activation with both sides of the septum, to the best of our knowledge, has not been previously described. Though intraseptal entrainment was demonstrated, definitive proof of participation of the entire pandiastolic signal recorded would require entrainment from each component with repeated overdrive sequences, which was not performed in this case. Recording of adjacent bystander components cannot be excluded. Entrainment from either RVOT or LVOT was not performed. The ability to record these presented signals requires cannulation of septal perforating vein in close proximity to or within the superior basal intraseptal substrate.

Prior studies have not demonstrated localized reentry completely confined within the intramural septum, which is the novelty of this report. These descriptions reiterated the propensity of LMNA to predominantly involve the septum,^5^^,^^9^ with Kumar and colleagues^3^ showing the majority of VT in lamin cardiomyopathy patients originating from the basal septum and exiting toward the left ventricle; however, intramural VT circuits were inferred rather than mapped via SPV. We have previously proposed a definition of localized reentry as >50% of diastole within <1.5 cm path.^10^ In this case, the entire diastolic phase was recorded from a single bipole pair.

Strategies such as substrate modification via transcoronary venous ethanol may warrant further consideration, particularly when a full-length septal scar is present on magnetic resonance imaging, which portends a poor prognosis.^11^ Although transcoronary venous ethanol ablation was attempted with limited efficacy by Kumar and colleagues^3^ in cases of VT refractory to multiple prior radiofrequency ablations, the midmyocardial VT circuits were not fully characterized. Further study to evaluate efficacy of transcoronary venous ethanol ablation in intramural VT,^8^ particularly when used in combination with complete mapping of the intramural circuit via coronary veins, should be considered.

Owing to high rates of recurrent VT associated with lamin cardiomyopathies, early collaboration and consultation with a heart failure team is warranted to discuss advanced management options, given modest outcomes from ablation and high risk of mortality from progression of heart failure. This case provides further mechanistic insights into the nature of septal VTs in this challenging patient population that may be entirely missed by traditional endocardial and epicardial mapping techniques.

## Conclusion

In midmyocardial septal scar substrates, SPV recordings may provide critical intramural information to elucidate the spatial dimensions and location of circuit-challenging VTs. Localized reentry recorded from a single bipole pair can be confined completely within the septum, which cannot be fully characterized from either endocardial surface.
